# Recessive *TBC1D24* Mutations Are Frequent in Moroccan Non-Syndromic Hearing Loss Pedigrees

**DOI:** 10.1371/journal.pone.0138072

**Published:** 2015-09-15

**Authors:** Amina Bakhchane, Majida Charif, Sara Salime, Redouane Boulouiz, Halima Nahili, Rachida Roky, Guy Lenaers, Abdelhamid Barakat

**Affiliations:** 1 Laboratoire de Génétique Moléculaire Humaine, Département de Recherche Scientifique, Institut Pasteur du Maroc, Casablanca, Morocco; 2 Université Hassan II Ain Chock, Laboratoire de Physiologie et génétique moléculaire, Km 8 Route d'El Jadida, B.P 5366 Maarif, Casablanca, 20100, Morocco; 3 Institut des Neurosciences de Montpellier, U1051 de l’INSERM, Université de Montpellier, BP 74103, 34091 Montpellier cedex 05, France; 4 PREMMi, Mitochondrial Medicine Research Centre, Université d'Angers, CHU Bât IRIS/IBS, Rue des Capucins, 49933 Angers cedex 9, France; Hadassah-Hebrew University Medical Center, ISRAEL

## Abstract

Mutations in the *TBC1D24* gene are responsible for four neurological presentations: infantile epileptic encephalopathy, infantile myoclonic epilepsy, DOORS (deafness, onychodystrophy, osteodystrophy, mental retardation and seizures) and NSHL (non-syndromic hearing loss). For the latter, two recessive (DFNB86) and one dominant (DFNA65) mutations have so far been identified in consanguineous Pakistani and European/Chinese families, respectively. Here we report the results of a genetic study performed on a large Moroccan cohort of deaf patients that identified three families with compound heterozygote mutations in *TBC1D24*. Four novel mutations were identified, among which, one c.641G>A (p.Arg214His) was present in the three families, and has a frequency of 2% in control Moroccan population with normal hearing, suggesting that it acts as an hypomorphic variant leading to restricted deafness when combined with another recessive severe mutation. Altogether, our results show that mutations in *TBC1D24* gene are a frequent cause (>2%) of NSHL in Morocco, and that due to its possible compound heterozygote recessive transmission, this gene should be further considered and screened in other deaf cohorts.

## Introduction

The *TBC1D24* gene (OMIM #613577) encodes a family of Tre2-Bub2-Cdc16 (TBC) domain containing RAB-specific GTPase-activating proteins, which are thought to be involved in exo- and endo-cytosis of vesicles [1, 2]. *TBC1D24* includes 8 exons, among which three: exons 3, 4 and 5 are alternatively spliced and exclusively present or absent in the mature messenger RNAs, thus leading to 4 isoforms [3, 4]. Expression of *TBC1D24* appears ubiquitous, with the exception of spleen, and is predominant in the developing brain, in chondrocytes from the distal phalanges, and in the scull [2, 3], thus explaining why *TBC1D24* mutations can induce epilepsies, mental retardation, onychodystrophies and osteodystrophies, as reported in the DOORS syndrome (OMIM #220500), or infantile myoclonic epilepsies (OMIM #605021) and epileptic encephalopathies (OMIM #615338). In addition, *TBC1D24* expression is also present in the cochlea in the inner and outer hair cells and in the spiral ganglion neurons [5, 6], substantiating also why *TBC1D24* mutations can induce hearing loss. In the last years, three publications reported mutations in *TBC1D24* associated to non-syndromic hearing loss (NSHL). Two recessive mutations, c.208G>T (p.Asp70Tyr) and c.878G>C (p.Arg293Pro) were identified in 4 consanguineous Pakistani families [5] (DFNB86; # 614617), and the c.533C>T (p.Ser178Leu) dominant mutation was simultaneously identified in a European family [7] and a Chinese family [6] (DFNA65; #616044).

We have recruited 136 Moroccan families with hearing impairment and performed genetic studies to identify the molecular determinants responsible for their disease [8–13].

Using whole exome sequencing (WES) on probands from recessive families without genetic diagnosis, we identified two pedigrees with compound heterozygote mutations in *TBC1D24*, which prompted further sequencing of all *TBC1D24* exons in the 62 families devoid of molecular genetic diagnosis. This led to the identification of an additional simplex case, again with compound heterozygote mutations.

## Patients and Methods

### Patients

**Fig 1:**
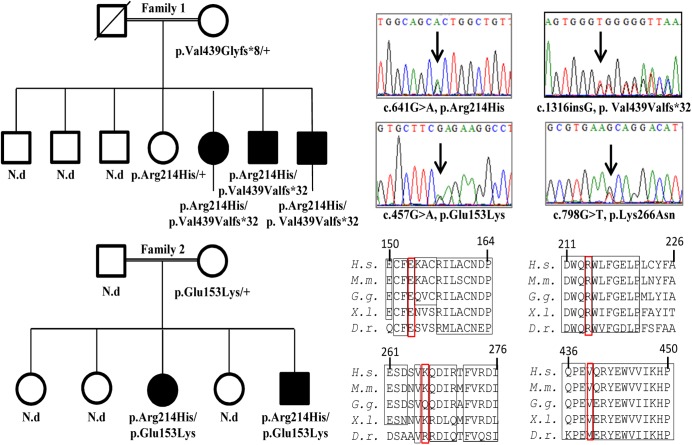
Mutations in *TBC1D24* segregate with non-syndromic hearing loss. (A) Pedigrees of the two families are shown with the segregation of the mutations identified in *TBC1D24*. (B) Electrophoregrams showing the 4 heterozygote mutations identified in this work. C) Alignments of TBC1D24 sequences around the 4 mutated amino acids highlighted by red rectangles (*H*.*s*.: *Homo sapiens*; *M*.*m*.: *Mus musculus*; *G*.*g*.: *Gallus gallus*; X.l.: *Xenopus laevis*; D.r.: *Danio rerio*).

Two unrelated Moroccan families were studied because of its parental consanguinity and the existence of two siblings with hearing loss. (Fig 1A) Audiological evaluation disclosed severe to profound congenital bilateral sensorineural hearing loss in all the affected members from these two families and in the simplex case from the third pedigree. Further clinical examination of the subjects disqualified any symptom or malformation that could be suggestive of a syndromic form of hearing loss. Before using WES, patient DNA were tested negative for *GJB2* mutation, mitochondrial (12sRNA) mutation and the 242G>A mutation in *LRTOMT*.

### Ethics statements

Written informed consent was obtained from all patients, and the genetic study was approved by the committee on research ethics of the Pasteur Institute of Morocco. This work was conducted according to the principles of the declaration of Helsinki.

### Whole Exome sequencing

Using the phenol chloroform method, genomic DNA was extracted from blood from affected and unaffected family members. Whole Exome Sequencing (WES) on the probands from family I and II was used to screen disease-causing variants, and performed at Otogenetics Corporation (Norcross, GA, USA). In brief, exome capture involved the use of the Agilent Human exome V5 (51Mb) capture kit, followed by paired end sequencing on a Hiseq2000 platform (Illumina, San Diego, USA). Reads were aligned against the human genome reference sequence hg19 (GRCh37), exome coverage obtained was higher than 47X, which provided sufficient depth to analyze variants. Candidate pathogenic variants were defined as missense, nonsense, splice-site and frameshift mutations with a minor allele frequency lower than 0.01, using the 1000 Genomes Project database and the Exome Variant Server (EVS).

To ascertain the segregation with the disease phenotype in these families, Sanger sequencing was performed to validate mutations in the candidate gene. Specific primers were designed using Primer3 (http://primer3.ut.ee/) (sequences are available upon request).

## Results and Discussion

Four novel variants in *TBC1D24* were identified and confirmed by Sanger sequencing. Segregation studies showed that all affected patients in family I and II were compound heterozygote for two distinct mutations, whereas unaffected ones were carrying one mutated allele or none (Fig 1A & 1B). Mutations were predicted to be pathogenic by SIFT, PolyPhen-2 and Mutation Taster softwares (Table 1). A common heterozygous variant, c.641G>A, p.Arg214His was present in the index patient from the two families and the simplex case. This mutation is referenced in NCBI database as rs200324356 and has a frequency of 0,2% in NCBI, 14/12736 in the Exome Variant Server database and 0.124% in ExAC browser. In addition, in family I (Fig 1A, top), we identified a heterozygous insertion c.1316insG leading to a frameshift (p.Val439Val.fs*32) truncating 120 amino acids of the protein. The two other affected members of this family were compound heterozygous for these mutations, whereas the unaffected brother and mother were heterozygote for the c.641G>A and the c.1316insG, respectively. In family II (Fig 1A, bottom), we identified another heterozygous mutation c.457G>A, leading to the p.Glu153Lys amino acid change. This mutation is referenced in NCBI database as rs376712059 and has a frequency of 1/12875 in the Exome Variant Server database. Sanger sequencing confirmed that the affected brother was compound heterozygous for the c.641G>A and c.457G>A mutations. In the simplex case, we identified another novel variant c.798G>T, leading to the p.Lys266Asn amino acid change, which is not referenced in any database. Segregation of this mutation was not achievable, because no other DNA sample from the family was available.

**Table 1:** Characteristics of the novel mutations identified in *TBC1D24* gene.

cDNA mutation	protein change	rs ID	NCBI	EVS	Sift	Polyphen2	Mutation Taster
c.457G>A	p.Glu153Lys	rs376712059		1/12875	0.29	1.0	56
c.641G>A	p.Arg214His	rs200324356	0.002	14/12736	0.4	0.997	29
c.798G>T	p.Lys266Asn	unknown			0.62	0.722	94
c.1316delT	p.Val439Val.fs32	unknown					

For each mutation, its position in the cDNA is given, as well as the amino-acid change, its reference number in NCBI database (rs ID), its frequency in NCBI and Exome Variant Server (EVS) database, and the predicted Sift, Polyphen-2 and Mutation Taster scores.

Thus, we have identified compound heterozygote mutations in *TBC1D24* in 3 Moroccan pedigrees with NSHL. All 4 mutations are proposed to be damaging by the Polyphen2 and Mutation taster prediction programs (Table 1), changing 3 amino-acids conserved among vertebrates, or introducing a frame shift deleting one fifth of the protein sequence (Fig 1C).

**Fig 2:**
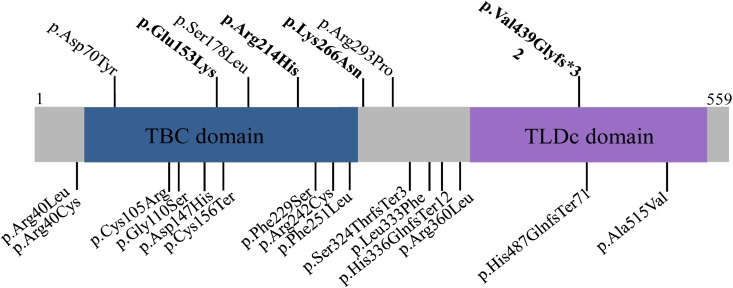
Schematic representation of the mutations responsible for non-syndromic hearing loss, in the TBC1D24 protein. The TDC and TLBc functional domains are represented on the TBC1D24 protein structure. The amino-acid changes identified in this work (in bold) and the published recessive and dominant mutations responsible for NHSL are shown on the top, while mutations responsible for DOORS syndrome, familial infantile myoclonic epilepsy, progressive myoclonus epilepsy and early-infantile epileptic encephalopathy-16 are shown below the protein structure.

The distribution of these mutations in TBC1D24 protein sequence (Fig 2), affects the TBC, the middle and the TLDc domains, thus suggesting that the integrity of these domains is crucial for TBC1D24 functions in the cochlea.

Importantly, the c.641G>A mutation was recurrently found, suggesting that it is a rather frequent polymorphism in the Moroccan population. This hypothesis is supported by a frequency of 1/1000 in the Exome Server Variant database and of 2/1000 in the NCBI database. Our analysis of a control Moroccan population showed that indeed this mutation can be considered as a frequent polymorphism, as we found it 4 times among 200 control DNA samples, thus with an allele frequency around 2%. In addition, we have not found a patient homozygous for this c.641G>A mutation, a situation that should be more frequent than the cases we reported here, associating the c.641G>A mutation (frequency: 0.02) to very rare mutations. Thus, although not biologically demonstrated, these observations suggest that the p.Arg214His mutation, by itself, has a mild deleterious effect on TBC1D24 functions, but leads to deafness when associated to another severe pathogenic mutation. In this respect, but without concrete evidence, we can hypothesize that this variant acts as a hypomorphic mutation, with probably no significant pathological consequence on hearing when homozygote. Our data also explain why, conversely to what we initially expected, we found compound heterozygote mutations in the 2 consanguineous families that we reported here.

The fact that we identified three families with *TBC1D24* compound heterozygote mutations is also an important finding of this work, as to date only consanguineous recessive pedigrees were identified, in addition to a single dominant mutation, thus suggesting that the association of two mutated *TBC1D24* alleles can induce NSHL. Furthermore the fact that mutations in *TBC1D24* have been found in 3 NSHL pedigrees out of 136 recruited in Morocco (2% of our index patients), promotes this gene as potentially crucial amongst other NSHL cohorts. Consequently, we much recommend that *TBC1D24* sequence is screened in other regions of the world, where causative mutations leading to inherited deafness have not been fully identified.
